# Communicative Nanomotors Reprogram Cancer Cell Death via Pyroptosis

**DOI:** 10.1002/anie.202510014

**Published:** 2025-07-02

**Authors:** Mingchen Sun, Luc van Oss, Chenxuan Wan, Daniela A. Wilson

**Affiliations:** ^1^ Institute for Molecules and Materials Radboud University Nijmegen Heyendaalseweg 135 Nijmegen 6525 AJ The Netherlands

**Keywords:** Cancer cell death, Cell fate reprogramming, Chemotaxis, Mitochondria‐targeting, Self‐assembled nanomotor

## Abstract

Nanomotors offer significant advantages over passive nanoparticles in biomedical applications. However, their potential has been largely restricted to cargo transport, with limited capacity for interaction with biological systems. Here, we present next‐generation self‐assembled nanomotors that not only exhibit chemotactic motility but also actively communicate with cells, reprogramming cell fate by inducing pyroptosis. These nanomotors are designed to respond to elevated reactive oxygen species (ROS) in the tumor microenvironment, triggering nitric oxide (NO)‐driven propulsion and selective mitochondria targeting via triphenylphosphine (TPP) surface engineering. This interaction induces mitochondrial damage, cytochrome c release, and activation of gasdermin E (GSDME)‐mediated pyroptosis. Furthermore, their chemotactic motility facilitates deeper tumor tissue penetration in 3D spheroids, demonstrating their ability to navigate physiological barriers. By shifting the paradigm from motility‐driven to interactive nanomedicine, this study establishes a transformative platform for targeted cancer therapy.

## Graphical abstract

Communicative nanomotors respond to elevated ROS in the tumor microenvironment, enabling active communication and selective mitochondrial targeting through non‐covalent surface engineering. Their precise interaction with cells induces mitochondrial damage, cytochrome c release, and pyroptotic cell death, while chemotactic motility ensures deeper tumor penetration, advancing intelligent cancer therapeutics.

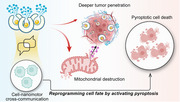

## Introduction

Nanomotors, self‐propelled nanoscale systems capable of converting various types of external energy into motion, have garnered significant interest in biomedicine due to their ability to navigate complex environments.^[^ ^1^, ^2^ ^]^ While extensive research has focused on enhancing propulsion mechanisms, their functionality has remained largely limited to transport‐based applications, such as drug delivery, imaging, and biosensing. For example, Liu et al. developed CaCO_3_ microparticles driven by GOx and a magnetic field to deliver doxorubicin, which was released in an acidic environment.^[^ ^3^ ^]^ Similarly, Song et al reported a light‐driven unsymmetrical nanomotor composed of copper‐rich Cu7S4 nanocrystals. This system exhibited directional motion and photothermal/photocatalytic properties, which facilitated transdermal penetration and disrupted bacterial membranes and DNA through light‐to‐heat conversion.^[^ ^4^ ^]^ Sanchez and coworkers demonstrated the swarming behavior of urease‐driven mesoporous silica nanoparticles for in vivo monitoring. By labeling these nanoparticles with ^124^I and ^18^F, they could be tracked using PET‐CT both in vitro and in vivo.^[^ ^5^ ^]^

Nanomotors have been fabricated from a wide range of materials including polymers, inorganic substances, and metals, and are powered by various stimuli such as light, magnetic fields, and chemical gradients.^[^ ^6^, ^7^, ^8^ ^]^ Significant efforts have been devoted to improving their motility, with faster speeds widely regarded as crucial for enhancing biomedical functionality. However, studies on speed and motility are typically performed in simplified buffer solutions under laboratory conditions, which do not adequately reflect the complexity of real physiological environments.^[^ ^9^, ^10^, ^11^ ^]^ Moreover, most nanomotors to date have primarily served as carriers for therapeutic or imaging agents, releasing their cargo by diffusion or stimulus‐triggered mechanisms at the target site. The next generation of nanomotor systems faces the critical challenge of advancing beyond mere transportation to meaningful interactions with biological environments and living systems. Such advancements would enable synthetic nanomotors to communicate with cells, modulate metabolic pathways, or even influence cell fate, enhancing their potential for more complex biomedical applications.

**Figure 1:**
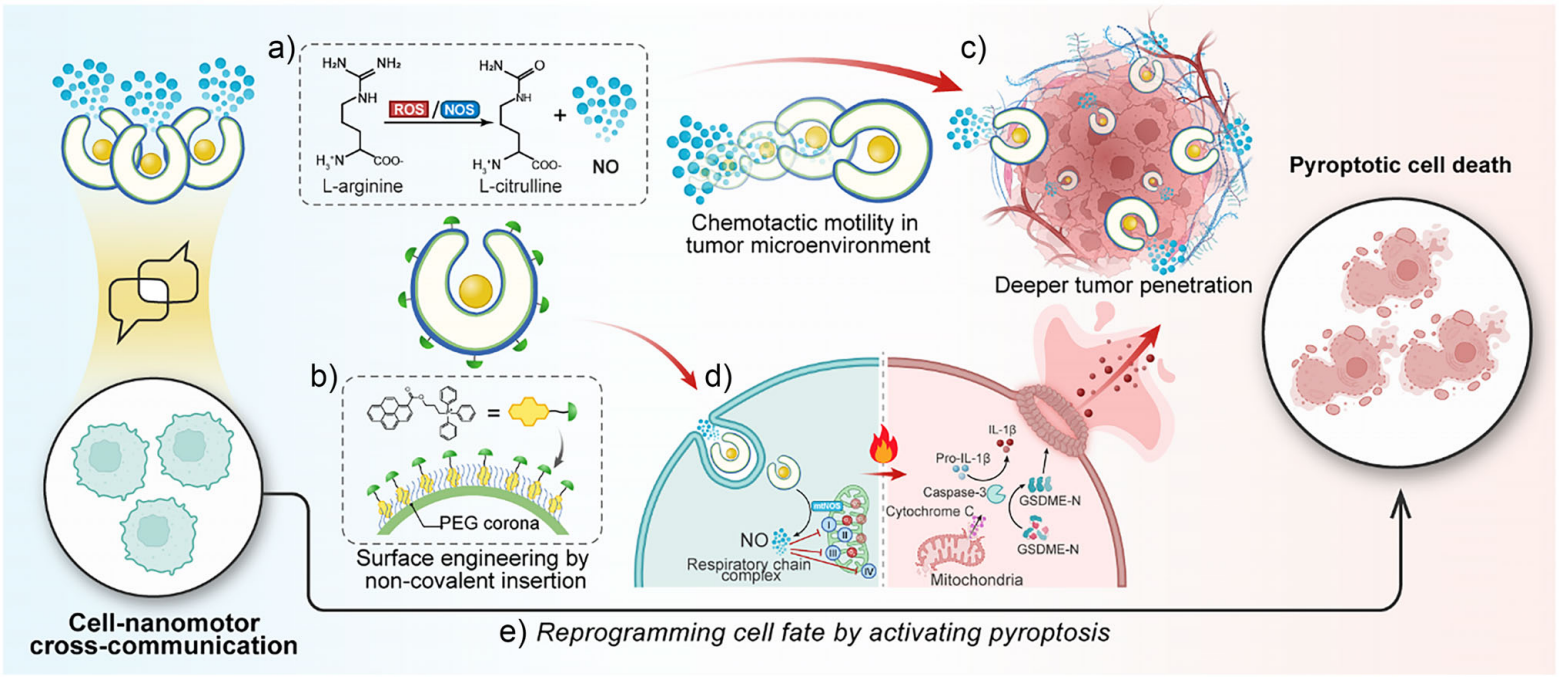
Schematic illustration of communicative nanomotors reprogramming cell death by inducing pyroptosis. a) Asymmetrical polymeric nanomotors propelled by the catalytic reaction between L‐arginine and ROS or NOS. b) Surface engineering with mitochondria moiety triphenylphosphine (TPP) through the recognition between PEG corona and pyrene. c) Chemotactic motility toward tumor microenvironment (TME) facilitates deeper tumor tissue penetration. d) and e) Intracellular NO leads to mitochondrial damage and cytochrome c release, shifting the mode of cancer cell death from non‐inflammatory death to pyroptosis.

In this study, we present a self‐assembled nanomotor system designed to actively communicate with and respond to tumor‐associated biochemical cues. Unlike conventional nanomotors that function solely as passive carriers, our system integrates chemotactic motility, mitochondrial targeting, and intracellular signaling to achieve responsive behavior within the tumor microenvironment. These nanomotors respond to reactive oxygen species (ROS stimuli) by generating NO, which not only propels their movement in complex physiological conditions but also induces targeted mitochondrial disruption and alteration of cell death pathway thus demonstrating the effective communication between the synthetic nanomotors and living systems. Surface anchoring of nanomotors with mitochondria targeting moiety triphenylphosphine (TPP) through the high‐affinity insertion of pyrene‐based TPP anchors onto the PEG corona, enables precise mitochondria localization, where NO‐mediated oxidative stress triggers cytochrome c release and subsequent activation of the pyroptotic cell death pathway.^[^ ^12^, ^13^, ^14^, ^15^, ^16^ ^]^ Specifically, encapsulated L‐arginine within the self‐assembled nanomotor is converted into nitric oxide (NO) by nitric oxide synthase (NOS), inducing mitochondrial damage and the release of cytochrome c.^[^ ^17^, ^18^ ^]^ This triggers a cascade involving caspase‐3 activation and gasdermin E (GSDME)‐mediated pyroptosis pathway, as confirmed by the pyroptotic‐like morphology and significant rise in extracellular inflammatory cytokines (IL‐1β) and lactate dehydrogenase (LDH). Furthermore, through the precise chemotactic motility on 3D Hela spheroids, our nanomotors can navigate through physical barriers, allowing this cross‐communication in deeper regions of the tumor tissue. Collectively, this work introduces the first‐of‐its‐kind interactive nanomotor system that goes beyond conventional cargo transport, not only senses and reacts to endogenous stimulus but also reprograms cell fate through targeted intracellular interactions. By demonstrating a new level of functional integration between synthetic materials and biological systems, this study opens exciting directions for next‐generation nanomotor design in biomedical applications (Figure 1).

## Results and discussion

### Design and Characterization of Communicative Nanomotors

**Figure 2:**
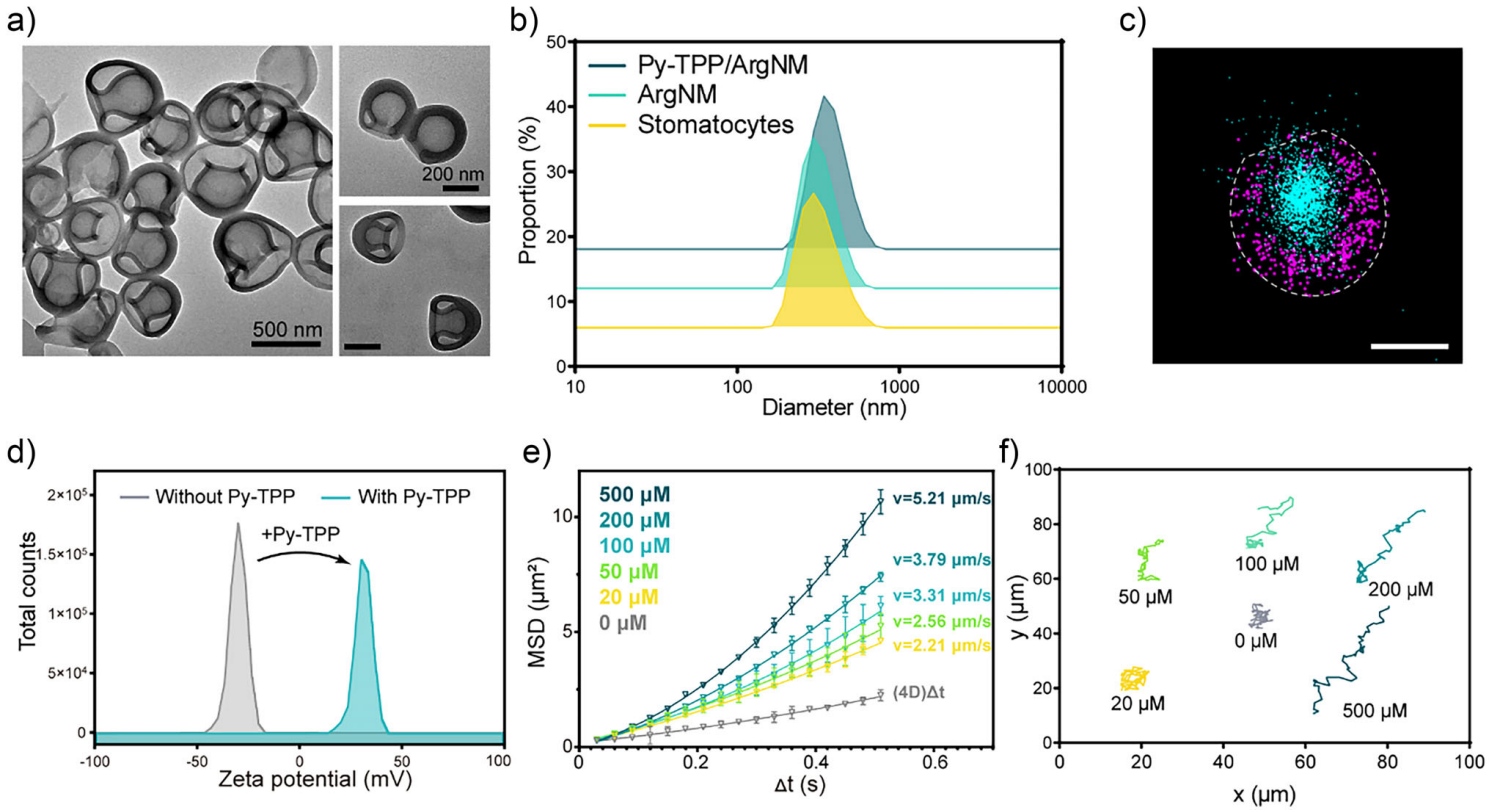
Preparation and characterization of nanomotors. a) TEM images of stomatocytes (left) and TPP functionalized stomatocytes (right). b) Size distribution of Py‐TPP functionalized arginine nanomotors (Py‐TPP/ArgNM), Arginine nanomotors (ArgNM), and stomatocytes. c) Super‐resolution fluorescence image of ArgNM encapsulated in stomatocyte. The polymeric surface was labeled by Py‐ATTO 488 (excitation: 488 nm, magenta) and the L‐Arginine nanoparticle was labeled by Alexa Fluor 647(excitation: 640 nm, cyan). Scale bar = 200 nm. d) Zeta‐potential of stomatocyte with and without Py‐TPP functionalization. e) Mean squared displacements (MSDs) of Py‐TPP/Arg in varying concentrations of H_2_O_2_. Velocity was extracted from the fitting of the average MSD of Py‐TPP/ArgNM, calculated from the tracking coordinates of, on average, 50 particles. f) Typical tracking paths of nanomotors, recorded for 2 s.

Self‐assembled stomatocyte nanomotors were synthesized from diblock copolymer poly(ethylene glycol)_44_‐*b*‐poly(styrene)_n_ (PEG‐PS) assembled polymersomes through controlled shape transformation under osmotic shock. The diameter of the polymersomes is about 396.9 nm with a well‐defined vesicular structure (Figure S1). A polyethylene glycol (PEG_2000_) solution was added to introduce instantaneous osmotic stress for the shape transformation from polymersomes into stomatocytes. By increasing the volume of the aqueous phase in the self‐assembly process from 1.0 mL to 1.3 mL, we induced greater rigidity and lower permeability in the polystyrene membrane.^[^ ^19^ ^]^ As a result, an equivalent amount of PEG_2000_ led to a lower osmotic stress over the membrane, decreasing the polymer chain mobility and restricting curvature changes, eventually forming stomatocytes with large openings.^[^ ^20^ ^]^ Transmission electron microscopy (TEM) confirmed a well‐defined vesicular structure (−396 nm) (Figure 2a). Arginine nanoparticles were prepared according to the literature procedure with a comparatively small particle size (−51 nm) (Figure S2).^[^ ^21^ ^]^ The structure of Arginine nanomotors (ArgNM) was characterized by a total internal reflection fluorescence (TIRF) microscope (ONI Ltd., UK). The encapsulated ATTO‐488 dye in the ArgNM emitted cyan fluorescence, as shown in Figure 2c and Figure S3. To visualize the polymeric structure, we utilized the specific interaction between the PEG corona and polycyclic aromatic hydrocarbon by inserting a pyrene‐Alexa Fluor 647 (AF647) conjugate onto the PEG corona, which emitted magenta fluorescence under a 640 nm laser.^[^ ^12^, ^13^ ^]^ Functionalization with triphenylphosphine (TPP) enabled specific mitochondrial targeting, while encapsulated L‐arginine served as an NO precursor. To confirm the successful TPP functionalization, we measured the zeta potential of functionalized nanomotors. Positively charged TPP altered the surface potential of ArgNM from −30 mV to around +30 mV (Figure 2d and Table S1). However, the surface functionalization generated negligible change in the particle size of the nanomotors (Figure 2b and Table S1). Next, nanoparticle tracking analysis was performed to study the real‐time movement of the nanomotors. In vivo, arginine is enzymatically converted to NO by nitric oxide synthase (NOS, including endothelial NOS, neuronal NOS, and mitochondrial NOS) and reactive oxygen species (ROS). In this study, we primarily used H_2_O_2_ to assess the motility of the nanomotors.

In Figure 2e Py‐TPP/ArgNM showed Brownian motion without H_2_O_2_, which was confirmed by the linear fitting of the mean squared displacement (MSD) curve.^[^ ^22^ ^]^ With increasing H_2_O_2_ concentration from 20 to 500 µM, the velocity rose from 2.21 µm s^−1^ to 5.21 µm s^−1^. The trajectories of Py‐TPP/ArgNM at varying concentrations of H_2_O_2_ are shown in Figure 2f. At 0 mM, only Brownian motion with negligible displacement was observed. Notable displacement occurred with higher H_2_O_2_ concentrations. The mechanism of nanomotor motility was studied by detecting the NO production in the medium considering the solubility of NO in water (Figure S4). As expected, with the increase of H_2_O_2_ concentration, the production rate of NO was remarkably accelerated, which is correlated with the rise in the movement speed. We also observed a concentration‐dependent consumption rate of arginine, reinforcing that the motility was driven by arginine converting to NO.

### Mitochondria‐Targeted Pyroptotic Cell Death

#### Cytochrome c Release Induced by Mitochondrial Damage

**Figure 3:**
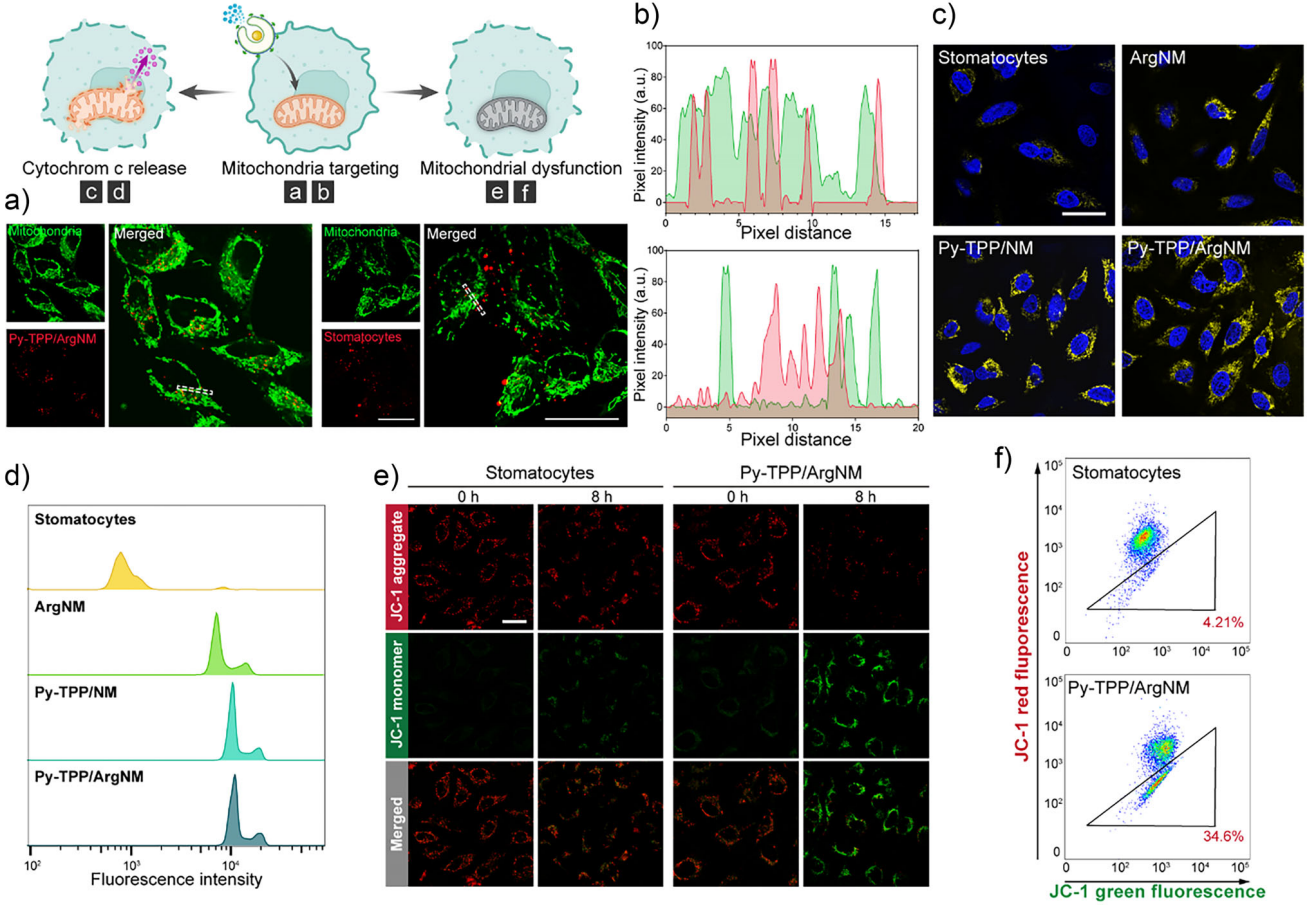
Cytochrome C release induced by mitochondrial damage. Py‐TPP directs the nanomotors to mitochondria, followed by distrut the mitochondria membrane, cytochrome c release, and mitochondria dysfunction. a) Colocalization of Py‐TPP/ArgNM or stomatocytes with mitochondria in Hela cells; scale bar = 20 µm. b) Fluorescence intensities of mitochondria and stomatocytes/Py‐TPP/ArgNM along the marked areas in a) analyzed by ImageJ. c) Fluorescence images showing the level and distribution of cytochrome c inside Hela cells upon different treatments. Yellow fluorescence and blue fluorescence represent cytochrome c and cell nucleus, respectively; scale bar = 20 µm. d) Fluorescence intensity of cytochrome c in Hela cells analyzed by flow cytometry. e), f) Mitochondrial membrane potential measurement by JC‐1 dye on Hela cells treated with stomatocytes and Py‐TPP/ArgNM; scale bar = 20 µm.

Confocal microscopy was used to assess the mitochondria targeting efficiency of the Py‐TPP functionalized nanomotors. Fluorescence imaging in Figure 3a showed that following a 6 h incubation with unfunctionalized stomatocytes, there was no overlap between the red and green fluorescence signals. In contrast, Py‐TPP functionalized nanomotors exhibited substantial colocalization with mitochondria (Figures 3b and S5), indicating that the Py‐TPP functionalization facilitated the targeted delivery of nanovesicles to mitochondria. Mitochondrial nitric oxide synthase (mtNOS), located on the mitochondrial membrane, catalyzes the conversion of L‐arginine to nitric oxide (NO) and L‐citrulline.^[^ ^23^, ^24^ ^]^ NO has been proven to inhibit the activity of respiratory chain complexes by both competing with oxygen and by nitrosylating or oxidizing mitochondrial components.^[^ ^25^ ^]^ Consequently, as depicted in Figure S6, treatment with Py‐TPP/ArgNM led to increased superoxide levels in Hela cells. Moreover, excessive peroxynitrite caused the opening of the permeability transition pore, resulting in the release of cytochrome c.^[^ ^26^ ^]^ The amount and activity of mtNOS can vary depending on factors such as hypoxia, cell/tissue type, and culture conditions. However, accurately determining mtNOS levels is challenging due to the small size and compact nature of mitochondria. Additionally, the intramitochondrial production of NO competes with extramitochondrial sources, making quantification difficult.^[^ ^27^ ^]^ Previous studies have indicated that cells with intact or damaged mitochondria contain the same amount of cytochrome c but in different locations. To assess cytochrome c release, we employed digitonin to permeabilize the cell membrane, allowing the release of cytoplasmic cytochrome c from the cells. Thus, the level of cytochrome C leakage is inversely correlated with the intensity of intracellular fluorescence.^[^ ^28^ ^]^ As shown in Figure 3c,d, cells treated with stomatocytes showed the highest cytochrome c content, as confirmed by the fluorescent intensity distribution given by flow cytometry. Conversely, a comparatively lower level of cytochrome c was observed in Py‐TPP/ArgNM treated cells, suggesting that Py‐TPP/ArgNM induced the release of cytochrome c. Additionally, increased concentration of Py‐TPP/ArgNM corresponded to enhanced cytochrome c release (Figure S7).

To further validate the mitochondrial damage caused by Py‐TPP/ArgNM, we used JC‐1 dye to assess the mitochondrial membrane potential. JC‐1 demonstrates potential‐dependent accumulation within mitochondria, manifested by a fluorescence emission shift from green (−525 nm) to red (−590 nm).^[^ ^29^ ^]^ As shown in Figure 3e, red fluorescence given by JC‐1 aggregate declined as the incubation time increased. Notably, after 8 h, JC‐1 emitted predominately green fluorescence, which further confirmed the mitochondrial damage caused by the nanomotors. Flow cytometry analysis reinforced these observations by showing a greater amount of JC‐1 monomers in Py‐TPP/ArgNM treated cells compared to those treated with stomatocytes (Figure 3f).

#### Chemotactic Tumor Penetration and Therapeutic Efficacy

**Figure 4:**
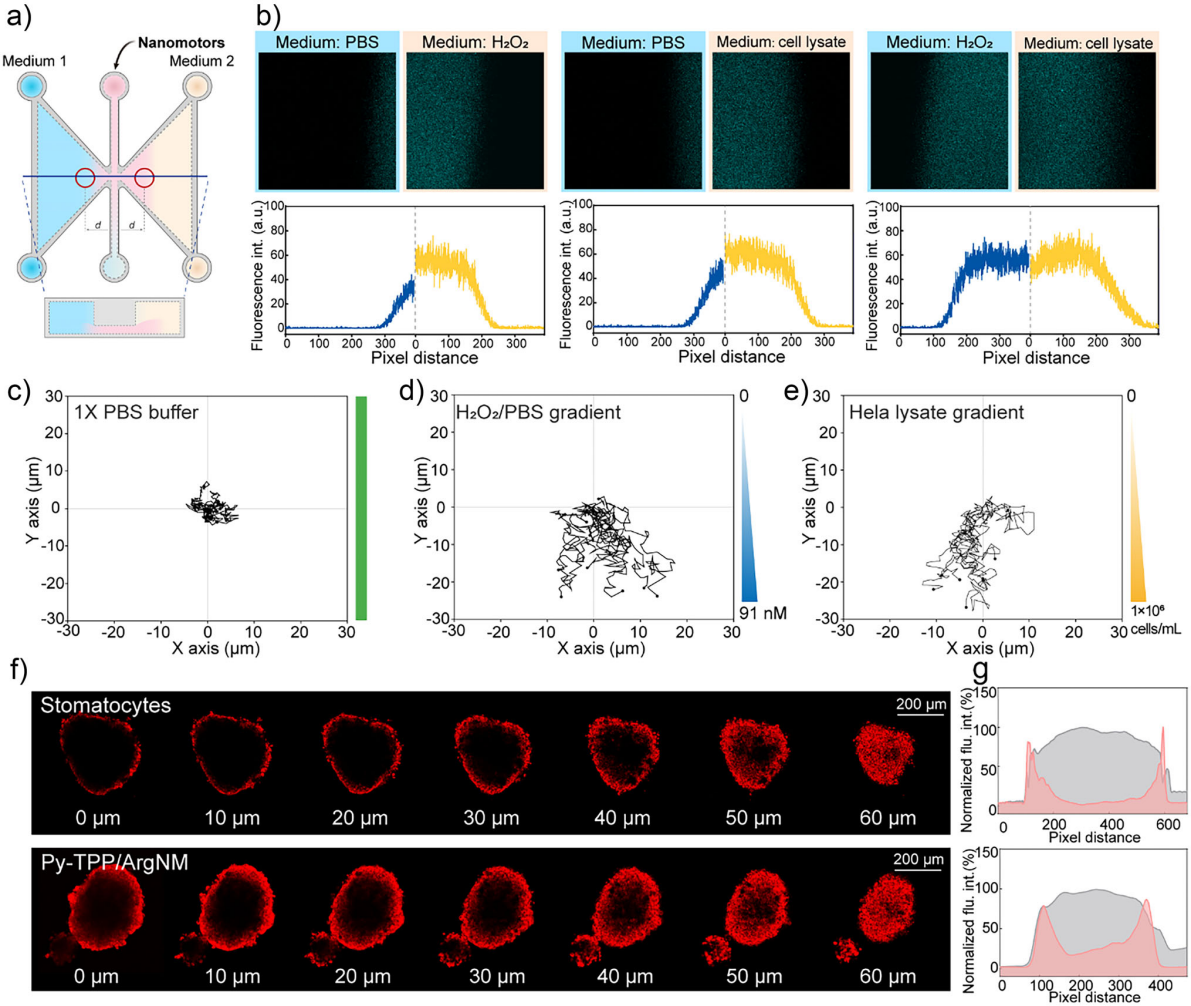
Chemotactic motility facilitates deeper tumor penetration. a) Schematic drawing of the μ‐Slide dishes from Ibidi. The suspension of Nile red labeled‐Py‐TPP/ArgNM (200 µg mL^−1^) was filled in the middle channel and two different media were added to the left and right chamber respectively. b) Fluorescent images were taken at the same distance from the center of the device. Line graphs reflect the average fluorescence intensity across each image. c) Tracking paths of the Py‐TPP/ArgNM over 50 consecutive frames (time interval = 0.33 s) in PBS buffer, H_2_O_2_ d), and Hela lysate (e). f) Z‐stack scanning of 3D HeLa spheroids after incubating with nile red labeled stomatocytes (300 µg mL^−1^) and Py‐TPP/ArgNM for 60 min. g) Line graphs illustrate the fluorescence distribution (red) across the 3D Hela spheroid at 90 min.

Increased metabolic activity of cancer cells, combined with oxidative stress, tumor‐associated inflammation, and local hypoxia, leads to high concentrations of hydrogen peroxide (H_2_O_2_) and reactive oxygen species (ROS) in the tumor microenvironment (TME). To simulate these conditions, we stimulated Hela cells with lipopolysaccharide (LPS) and used the cell lysate (1 × 10^6 cells mL^−1^) as a model for the TME.^[^ ^30^ ^]^ First of all, we examined the motility of our nanomotor in these two conditions. As shown in Figure S8, in 114.5 nM of H_2_O_2_ the nanomotors reached a velocity of 1.70 µm s^−1^, comparable to that in Hela lysate (1.69 µm s^−1^). As illustrated in Figure 4a, the μ‐Slide Chemotaxis (ibidi) dishes were used to study the tumor penetration ability of the nanomotors, where the left and right chambers were filled with different mediums. Additionally, an H_2_O_2_ solution with equivalent oxidizing ability was adopted as a positive control (Figure S9; Tables S3 and S2). When the left chamber contained PBS buffer and the right chamber contained H_2_O_2_, the motor showed significantly increased motility in the right chamber, leading to a larger fluorescence area in the image (Figure 4b). Moreover, Py‐TPP/ArgNM showed similar motion activity in the Hela cell lysate. Intriguingly, when both chambers were filled with H_2_O_2_ and cell lysate of equivalent oxidizing capacity, the nanomotors diffused to the same distances. This observation demonstrated that Py‐TPP/ArgNM possesses active motion behavior, particularly in an oxidative environment with elevated levels of reactive oxygen species (ROS). As shown in Figure 4c, Py‐TPP/ArgNM showed inactive Brownian motion in homogeneous Pbs buffer, while in H_2_O_2_ and Hela lysate gradients (diluted with Pbs) the Py‐TPP/ArgNM collectively moved to the high concentration (Figure 4d,e). Next, we employed 3D Hela spheroids to examine the tumor tissue penetration of Py‐TPP/ArgNM.^[^ ^31^ ^]^ The fluorescence images in Figure S10 show the fluorescence distribution of Nile red‐labeled stomatocytes or Py‐TPP/ArgNM across the tumor spheroids. In Figure 4f, Z‐stack scanning images revealed that after a 60 min incubation, Py‐TPP/ArgNM penetrated deeper into the tumor spheroid compared to stomatocytes. The accompanying line graphs further show that at 90 min, the fluorescence of Py‐TPP/ArgNM was more concentrated in the center of the tumor spheroid compared to that of stomatocytes (Figure 4g).

### In Vitro Hela Cell Pyroptotic Death

**Figure 5:**
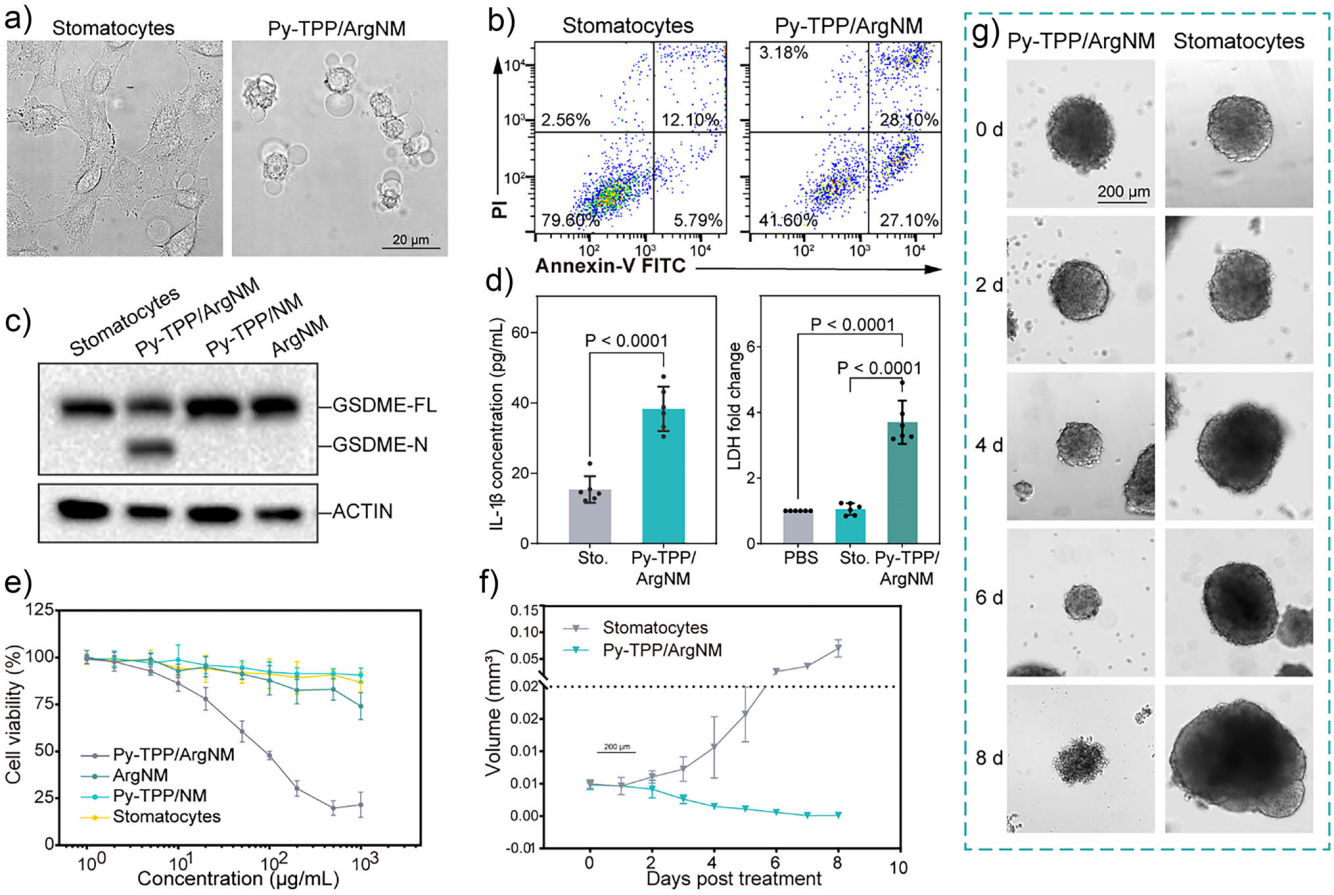
Motile nanomotors induce Hela cell pyroptotic death in vitro. a) Bright‐field images of Hela cells incubated with stomatocytes and Py‐TPP/ArgNM for 6 h. Black arrows indicate the cells showing a pyroptotic morphology; scale bar = 20 µm. b) Cell death nature of Hela cells treated with Py‐TPP/Arg/NM analyzed by flow cytometry with PI and Annexin V‐FITC staining. c) Immunoblots of GSDME cleavage of Hela cells treated with different vesicles. GSDME‐FL: full‐length of GSDME (for uncropped gel: Figure S13). d) Quantification of IL‐1β in the supernatant and LDH release after treating with stomatocytes or Py‐TPP/ArgNM. e) CCK‐8‐based cell viability of Hela cells treated with different nanovesicles for 72 h. f) Daily volume change of 3D Hela spheroid after treating with stomatocytes and Py‐TPP/Arg/NM. Representative images are taken on Day 0 and Day 8. g) Representative images of 3D HeLa spheroids treated with Py‐TPP/ArgNM and blank stomatocytes; scale bar = 200 µm.

Pyroptosis is morphologically defined by cell swelling and rupture of the plasma membrane, resulting in the release of pro‐inflammatory cytokines and cellular contents into the extracellular space. We first examined the morphological changes in the cells. Hela cells treated with Py‐TPP/ArgNM exhibited pronounced cell swelling and the formation of large bubbles on the plasma membrane, which are hallmark features of pyroptosis (Figure 5a). This was further confirmed by flow cytometry analysis using Annexin V‐FTIC and propidium iodide (PI) co‐staining. As shown in Figure 5b, the percentage of Annexin V‐FITC and PI double‐positive cells (Annexin V^+^ and PI^+^) increased from 12.10% to 28.10% after treatment with Py‐TPP/ArgNM for four hours. The Gasdermin family proteins have been recognized as the executors of pyroptosis. The N‐terminal of gasdermin proteins is liberated by the caspase proteases cleaving the middle linker of gasdermins proteins and subsequently forming pores across the cell membrane.^[^ ^16^ ^]^ Hence, fragments of gasdermin proteins are one of the most studied markers for pyroptosis. The western blotting assay in Figure 5c showed distinct GSDME N‐terminal expression following treatment of Py‐TPP/ArgNM, whereas no such expression was observed in response to the treatment with blank stomatocytes, Py‐TPP/NM, or ArgNM. Moreover, cell pyroptotic death is also defined by the secretion of pro‐inflammatory cytokines such as interleukin‐1β (IL‐1β) and lactate dehydrogenase (LDH).^[^ ^32^, ^33^ ^]^ Concentrations of IL‐1β in the cell culture medium increased significantly (15.4 ± 3.8 versus 38.4 ± 6.3, pg mL^−1^, stomatocytes versus Py‐TPP/ArgNM, *p* < 0.0001), as did LDH levels (1.1 ± 0.2 versus 3.7 ± 0.7, multiples of changes to PBS, stomatocytes versus Py‐TPP/ArgNM, *p* < 0.0001) (Figure 5d). The overall cytotoxicity of Py‐TPP/ArgNM was evaluated by CCK‐8 assay over 72 h. As shown in Figure 5e, Py‐TPP/ArgNM exhibited outstanding cytotoxicity at 50 µg mL^−1^ (calculated as the mass concentration of PEG‐PS), with cell viability decreased to 60.63%. At the concentration of 500 µg mL^−1^, only 19.86% of cells remained viable. By comparison, ArgNM showed minor cytotoxicity with concentrations of 200 µg mL^−1^ and higher due to the non‐specific S‐nitrosation effect of NO. Regarding biocompatibility, NIH‐3T3 cells remained >90% viable when the concentration was 1000 µg mL^−1^, suggesting good in vitro biocompatibility (Figure S11). To evaluate the nanomotors’ ability to inhibit tumor growth, we incubated Py‐TPP/ArgNM with 3D Hela spheroids and observed their growth (Figure 5f,g). The medium and nanoparticles were refreshed every 24 h. The initial diameter of tumor spheroids was around 100 µm. However, on the 8^th^ day of incubation with Py‐TPP/ArgNM, the size of the tumor spheroid was found to reduce significantly. In contrast, Hela spheroid treated with stomatocytes continued growing uncontrollably (0.00011 ± 0.0001 versus 0.07 ± 0.016, mm^3^, Stomatocytes versus Py‐TPP/ArgNM, *p* < 0.001).

## Conclusion

This work pioneers a shift from passive nanomedicine to interactive motile systems capable of engaging with cellular environments. By integrating chemotactic motility, mitochondria targeting, and NO‐mediated pyroptosis induction, these nanomotors establish a new paradigm for nanotherapeutics. The self‐assembled nanomotors demonstrate the ability to communicate and interact with biological systems, effectively instructing changes in cancer cell states. Surface engineering with TPP enabled precise mitochondria targeting, while chemotactic motility in the tumor microenvironment—modeled using Hela cell lysate—ensured effective tumor navigation. In 3D Hela spheroids, the nanomotors exhibited enhanced tissue penetration within 60 to 90 min, successfully delivering NO to mitochondria. This led to mitochondrial damage, cytochrome c release, and GSDME‐mediated pyroptotic cell death, as confirmed by characteristic cell morphology, GSDME‐N expression, and inflammatory cytokine secretion. Furthermore, Py‐TPP/ArgNM displayed remarkable anticancer efficacy in both monolayer and 3D Hela spheroids. By effectively reprogramming the cell death pathway to pyroptosis, our motile nanomotors overcome physical and immunological barriers in solid tumors. This study offers a promising platform for inducing pyroptosis in innovative cancer therapies, addressing both physical and immunological barriers in solid tumors. However, we also recognize the possibility of off‐target effects, especially since elevated ROS levels and NOS are not unique to tumor tissues and cancer cells. Unintended activation of Py‐TPP/ArgNMs may also occur in inflamed or other pathologically oxidative environments. Further optimization of the nanomotor design, along with comprehensive in vivo studies, will be essential to improve specificity and ensure controlled activation in target tissues.

## Author Contributions

M.S. and D.A.W. conceived the concept. M.S. designed and performed the experiments, interpretated the results, and prepared the first draft of the manuscript. L.v.O. performed the characterization of the nanomotors. C.W. performed experiments on cytochrome c release. All authors took part in the discussion and assisted in preparing the final version of the manuscript.

## Conflict of Interests

The authors declare no conflict of interest.

## Supporting information



Supporting Information

## Data Availability

All data needed to evaluate the conclusions in the paper are present in the paper and/or the Supplementary Information. The data that support the findings of this study are available from the corresponding author upon reasonable request.
