# Supplementary material for: Communicative Nanomotors Reprogram Cancer Cell Death via Pyroptosis
Source: Angew Chem Int Ed Engl. 2025 Jul 2;64(34):e202510014. doi: 10.1002/anie.202510014 (PMC12363611; doi:10.1002/anie.202510014)
Supplement: Supplementary file 1 — Supporting Information [file ANIE-64-e202510014-s001.pdf]

Communicative Nanomotors Reprogram Cancer Cell Death via Pyroptosis

Mingchen Sun[1], Luc van Oss[1], Chenxuan Wan[1], Daniela A. Wilson[1,*]

[1]Radboud University Nijmegen, Institute for Molecules and Materials, Heyendaalseweg 135, 6525 AJ, Nijmegen, The Netherlands

Corresponding author e-mail: d.wilson@science.ru.nl

Content

Experimental section **2**

Chemicals 2

Preparation of polymersomes and stomatocytes 2

Preparation of ultra-small arginine nanoparticles 2

Surface engineering of Py-TPP onto PEG corona 3

Polymersome diameter 3

Motility study 3

Cell culture 4

Mitochondria targeting and destruction 4

Deeper tumor penetration 5

Pyroptotic cell death 5

Anti-tumor capacity in vitro 6

Statistical Analysis 6

Supplementary figures **7**

Experimental section

Chemicals

All reagents and chemicals were purchased from commercial sources and used as received. For Preparation of nanomotors: tetrahydrofuran (THF, VWR International), 1,4-dioxane (Biosolve), Nile red (Thermo Fisher Scientific), PEG $_{2k}$ (Sigma-Aldrich), L-Arginine (Sigma-Aldrich), Poly(D,L-lactide-co-glycolide) (Sigma-Aldrich Chemie), α-naphthol (TCI Europe NV), 1-(bromoethyl)pyrene (Sigma-Aldrich Chemie). For in vitro studies: Hela cells (The American Type Culture Collection, ATCC), Dulbecco's Modified Eagle Medium (DMEM) and Fetal Bovine Serum (FBS) (Thermo Fisher Scientific), 4% Paraformaldehyde and anti-cytochorme C Alexa Fluor 647 (Fisher Scientific), JC-1 (Bio Connect B.V.), FITC-Annexin V and Annexin V binding buffer (BD Pharmingen), MitoTrack 488 Dye (Sigma-Aldrich Chemie), Human IL-1 (Interleukin 1 Beta) ELISA Kit and Lactate dehydrogenase (LDH) Activity Assay Kit (Sanbio B.V.), Invitrogen™ MitoSOX™ Mitochondrial Superoxide Indicators (Thermo Fisher Scientific), Triton X-100 lysis buffer (Fisher Scientific).

Preparation of polymersomes and stomatocytes

10 mg of PEG_{200}-*b*-PS_{44} was dissolved in a mixture of tetrahydrofuran (THF) and dioxane (4:1, v/v) in a 15 mL vial with a stirring bar. After stirring for 30 min, 1.3 mL of Milli-Q water was added to the solution at a rate of 1 mL/h at room temperature under vigorous stirring. For shape transformation, 200 μL of the polymersome suspension was transferred to a 0.5 mL Eppendorf, and 20 μL of PEG $_{2000}$ aqueous solution (10 mg/mL) was added, followed by vigorous stirring for another 5 min. Afterwards, approximately 2.5 mL of Milli-Q water was promptly mixed with the suspension to quench the structure. The organic solvent was removed by repeated centrifugation. Die

Preparation of ultra-small arginine nanoparticles

The method for preparing the ultra-small arginine nanoparticles was adapted from previous reports. Briefly, 12 mg of arginine was dissolved in 2 mL of a mixture of water and ethanol. 12 mg of poly(lactic-co-glycolic acid) was dissolved in 2 mL of THF and was added to the arginine solution dropwise in a water-bath ultrasonication. Then the organic solvent was removed by rotary evaporation. The drug loading and encapsulation efficiency was determined by the chromogenic reaction between L-Arginine and α-naphthol.

Surface engineering of Py-TPP onto PEG corona

Py-TPP was synthesized by a method from a previous study. 1-Pyrenecarboxylic acid (74 mg, 0.3 mmol) and K_2CO_3 (55 mg, 0.4 mmol) were dissolved in 3 mL of THF and stirred at room temperature for 30 minutes under N_2. 3-Bromopropyl triphenylphosphonium bromide (97 mg, 0.2 mmol) was added to the reaction mixture and stirred overnight under N_2 at room temperature. The crude product was purified by column chromatography on silica gel (heptane: ethyl acetate: ethanol = 20:56:24). For the surface functionalization of nanomotors, an aqueous solution of Py-TPP was mixed with the suspension of nanomotor at a concentration of 1 mg/mL (final concentration of Py-TPP: 20 μM). After leaving it at room temperature for 10 min, the mixture was centrifuged at 14000 rpm for 10 min to remove the excessive Py-TPP. The surface functionalized nanomotors were resuspended with a certain amount of water for further use.

Polymersome diameter

Dynamic light scattering (DLS) measurements were carried out at 25 °C using MalvernZetasizer Nano-ZS (Malvern Instruments) equipped with a He-Ne laser (633 nm, 4 mW) and Avalanche photodiode detector (173°) to evaluate the average hydrodynamic diameter (D_h) and polydispersity (PDI) of stomatocytes. DLS uses the fluctuations in scattered light intensity due to the Brownian movement of the particles to determine their size distribution derived from the Stokes-Einstein equation by assuming a hard sphere model.

Motility study

The movement of the nanomotors was evaluated at a series of concentrations of H_2O_2 (100 mM, 50 mM, 20 mM, 10 mM, and 5 mM). Nanoparticles (around 1×10^9 particles per mL) were injected into the sample chamber of NanoSight LM10 after being mixed with different concentrations of H_2O_2. The motion behavior of particles was analyzed with nanoparticles tracking software (NTA 2.2) using video recorded by NanoSight (30 s per video, 30 frames per second). Hela cells were treated with 10 ng/mL lipopolysaccharide (LPS) for 4 hours and then collected at a density of 10^3, 10^4, 10^5, and 10^6 cells/mL. The cell suspensions were then incubated with ice-cold lysis buffer for 30 min at 4°C. After centrifuging at 12000 rpm for 20 min, the Hela cell lysate was obtained and stored at -20°C for further use. Nile red labeled Py-TPP/ArgNM suspension (200 μg/mL) was injected into the middle channel of μ-Slide Chemotaxis (ibidi), followed by filling the left and right chambers with different mediums (either PBS buffer, or Hela cell lysate containing 10^6 cells/mL, or H_2O_2). Confocal microscopy

was used to observe the edge of fluorescence given by Nile red labeled Py-TPP/ArgNM. Fluorescence images were taken at the same distance from the center of the device.

Cell culture

All cells were purchased from the American Type Culture Collection (ATCC). Hela Cells and NIH/3T3 cells were cultured in DMEM containing 10% fetal bovine serum (FBS) and antibiotics (penicillin 100 U/mL and streptomycin 100 μg/mL) at 37°C with 5% CO_2. Trypsin/EDTA was used to digest cells.

Mitochondria targeting and destruction

Hela cells were seeded in an 8-well plate (ibidi GmbH) with a density of 5×10^4 per well and incubated overnight for adherence. Stomatocytes and Py-TPP/NM were incubated with cells for another 6 h. After PBS rinsing, mitochondria dye staining (100 nM, 20 min), formaldehyde fixation (4% paraformaldehyde, 15 min), and fluorescent images were obtained by confocal fluorescence microscopy with a 40×objective. The colocalization of mitochondria and nanomotors was analyzed with ImageJ. To determine the cytochrome C release. Hela cells were treated with different nanovesicles and resuspended in 100 uL of ice-cold permeabilization buffer containing 10 mM KCl and 125 μg/ml digitonin. The mixture was then incubated on ice for 3-5 min. After fixing with 4% paraformaldehyde and washing with ice-cold PBS buffer, cells were further incubated with anti-cytochrome C Alexa Fluor 647 at 4°C overnight. The fluorescence emitted by anti-cytochrome C Alexa Fluor 647 was measured by a BD flow cytometry using the propidium iodide channel. Fluorescent images were obtained by confocal microscopy using the DAPI and Nile red channel. For determining the mitochondrial membrane potential, after incubating with Stomatocytes and Py-TPP/NM for 4 and 8 h, Hela cells were rinsed with prewarmed PBS three times. JC-1 working solution was added to each well (final concentration: 15.3 μM, 10 μg/mL) and incubated at 37°C for 15 min. Confocal fluorescence microscopy was used to observe the potential of the mitochondria membrane using both FITC and Nile red channels. For flow cytometry analysis, Hela cells were digested and centrifuged at 1200 rpm for 4 min. Cells were resuspended with JC-1 working solution (10 μg/mL) and incubated for 15 min at 37°C. After washing three times with PBS, the fluorescence of JC-1 in mitochondria was analyzed by a BD flow cytometer using FITC and propidium iodide channel.

Deeper tumor penetration

Multicellular 3D Hela spheroids were cultured using an ibidi 8-well dish coated with Bioinert surface. Hela cells were seeded with a density of 1000 cells/mL and then were placed in a cell incubator for 5 days. Cell spheroids that reached a certain size were selected for the following experiments. Hela spheroids with a diameter of about around 300 μm were selected and treated with stomatocytes and Py-TPP/ArgNM. The Z-stack mode of the confocal microscopy was used to observe the fluorescence distribution of Nile red at 30, 60, and 90 min (from top to center, interval = 10 μm). ImageJ was used to analyze the fluorescent distribution in tumor spheroid.

Pyroptotic cell death

Hela cells were seeded in an 8-well plate (ibidi GmbH) with a density of 5×10^4 per well and incubated overnight for adherence. Stomatocytes and Py-TPP/NM were incubated with cells for another 4 h. A confocal microscopy was used to observe the morphology change of Hela cells. Cells were then digested and stained with PI and Annexin-V FITC and analyzed by flow cytometry. For western blot analysis, cells with different treatments were lysed in buffer containing 25 mM tris(hydroxymethyl) aminomethane (Tris, pH 7.4), 150 mM NaCl, 0.5% sodium deoxycholate, 0.1% SDS buffer, and 1% Triton X-100. The cell lysate was clarified by centrifuging at 12000 rpm for 10 min at 4°C, and the supernatant was diluted with 4×loading buffer, followed by boiling for 10 min. Protein samples (20 μg) were fractionated by sodium dodecyl sulfate-polyacrylamide gel electrophoresis and transferred to polyvinyl difluoride membranes with a Mini-PROTEAN Tetra system (Bio-Rad). Blots were then blocked with 5% skim milk in Tris-buffered saline plus 0.05% Tween-20 and probed with appropriate antibodies according to the manufacturer's instructions. The concentration of IL-1β in cell supernatants was detected by enzyme-linked immunosorbent assay (ELISA) using a commercial kit. For the sample preparation, Hela cells were seeded in a 6-well plate with a density of 5×10^4 per well. After overnight incubation, TPP/ANM and ANM were added and incubated for another 6 hours. Cell culture medium was centrifuged (5000 rpm, 20 min) at 4°C before measurement. The LDH activity assay was performed according to the manufacturer's instructions. The absorbance of each well was measured at 450 nm. All measurements were performed six times in parallel.

Anti-tumor capacity in vitro

Hela cells were seeded in an 8-well plate (ibidi GmbH) with a density of 5× 10^4 per well and incubated overnight for adherence. Different nanovesicles at a series of concentrations (1, 2, 5, 10, 20, 50, 100, 200, 500, 1000 μg/mL, calculated as PEG-PS) were incubated with the cells for 72 h. The nanovesicles were refreshed every 24 hours. Afterwards, a CCK-8 assay was performed to evaluate the cell viability using the Spark M10 plate reader at 450 nm. 3D Hela spheroids with a diameter of around 300 μm were transferred to a new dish and incubated with different nanovesicles. The nanovesicles were refreshed every 24 hours and the size was measured and recorded every day.

Statistical Analysis

All quantitative experiments were analyzed for statistical significance between the sampled conditions. The statistical tests used (Student's t-test for two groups, one-way analysis of variance (ANOVA) test, and Tukey's honestly significant difference (HSD) post hoc test for more than two groups) are defined in the corresponding figure legends for all panels. Statistical significance is shown as follows: 0.01 < *P < 0.05; 0.001 < **P < 0.01; ***P <0.001, NS = not significant (P > 0.05).

Supplementary figures

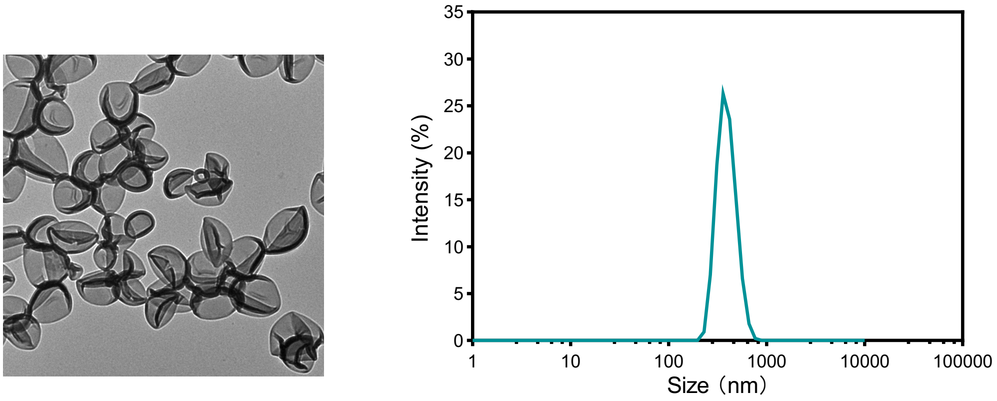

Figure S1. TEM image (up) and size distribution of PEG$_{44}$-*b*-PS$_n$ polymersomes.

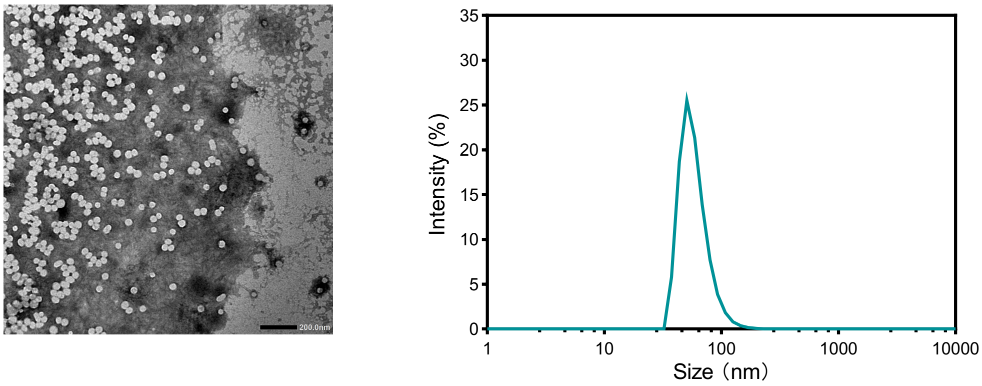

Figure S2. TEM image (up) and size distribution of Arg-PLGA nanoparticles (ArgNM)

Table S1. Diameter and Zeta-potential of nanovesicles.

	Diameter			Zeta-potential	
	**Diameter (nm)**	**Mean±SD**	**PDI**	**Zeta-potential (nm)**	**Mean±SD**
**Stomatocytes**	376.75	396.90±<br>17.55	0.09	-33.56	-31.02±2.32
	405.10		0.14	-29.01	
	408.85		0.11	-30.50	
**ArgNM**	46.63	51.92±<br>6.49	0.21	NA	
	49.95		0.25		
	59.16		0.19		
**Py-TPP/ArgNM**	378.73	377.76±<br>2.83	0.10	27.35	30.36±2.61
	374.58		0.14	31.99	
	379.98		0.08	31.74	

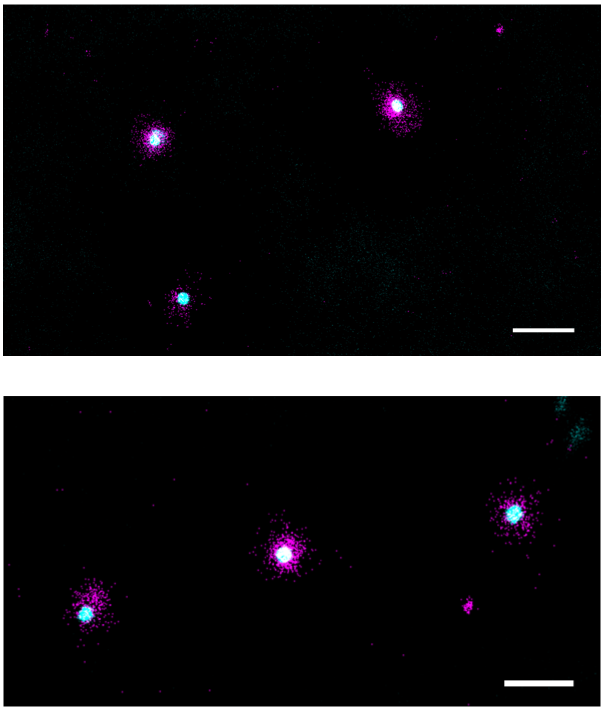

Figure S3. Supplementary super-resolution fluorescence images of ArgNM encapsulated in stomatocytes. The polymeric surface was labeled by Py-ATTO 488 (excitation: 488 nm, magenta) and the L-Arginine nanoparticle was labeled by Alexa Fluor 647(excitation: 640 nm, cyan). Scale bar = 500 nm.

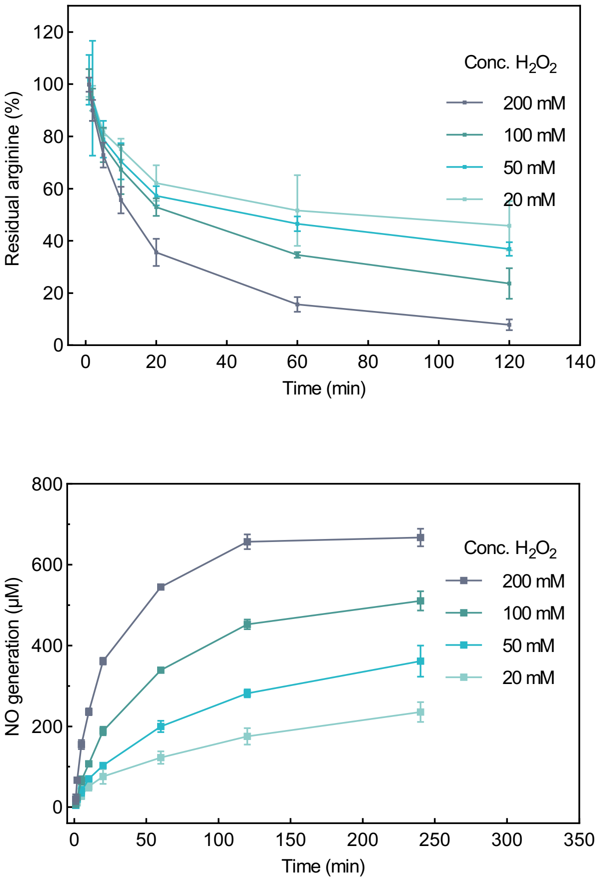

Figure S4. Arginine consumption (up) and NO generation (down) in different concentrations of H_2O_2.

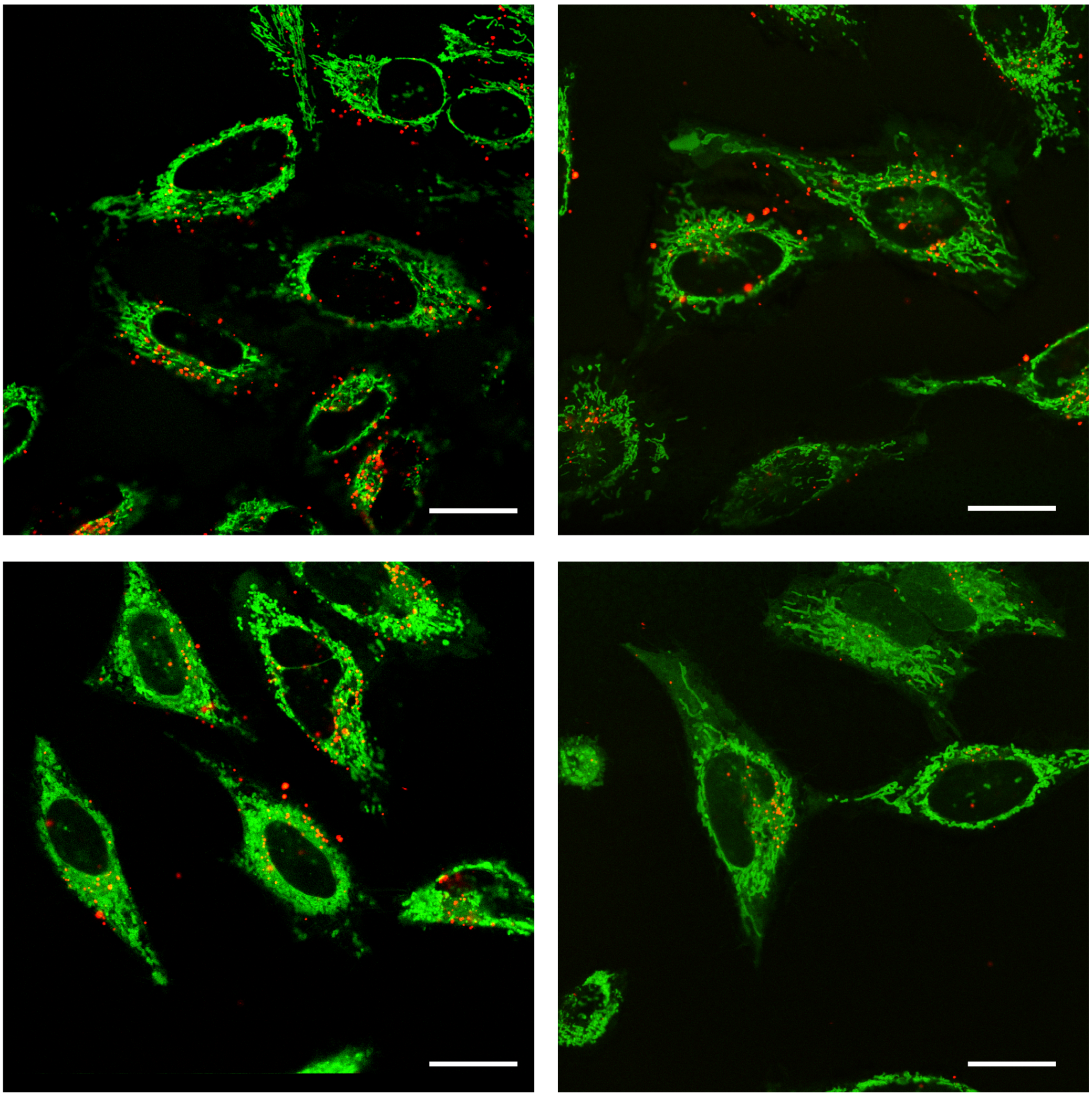

Figure S5. Supplementary images showing the colocalization of Py-TPP/ArgNM (red fluorescence) and mitochondria (green fluorescence); scale bar = 10 µm.

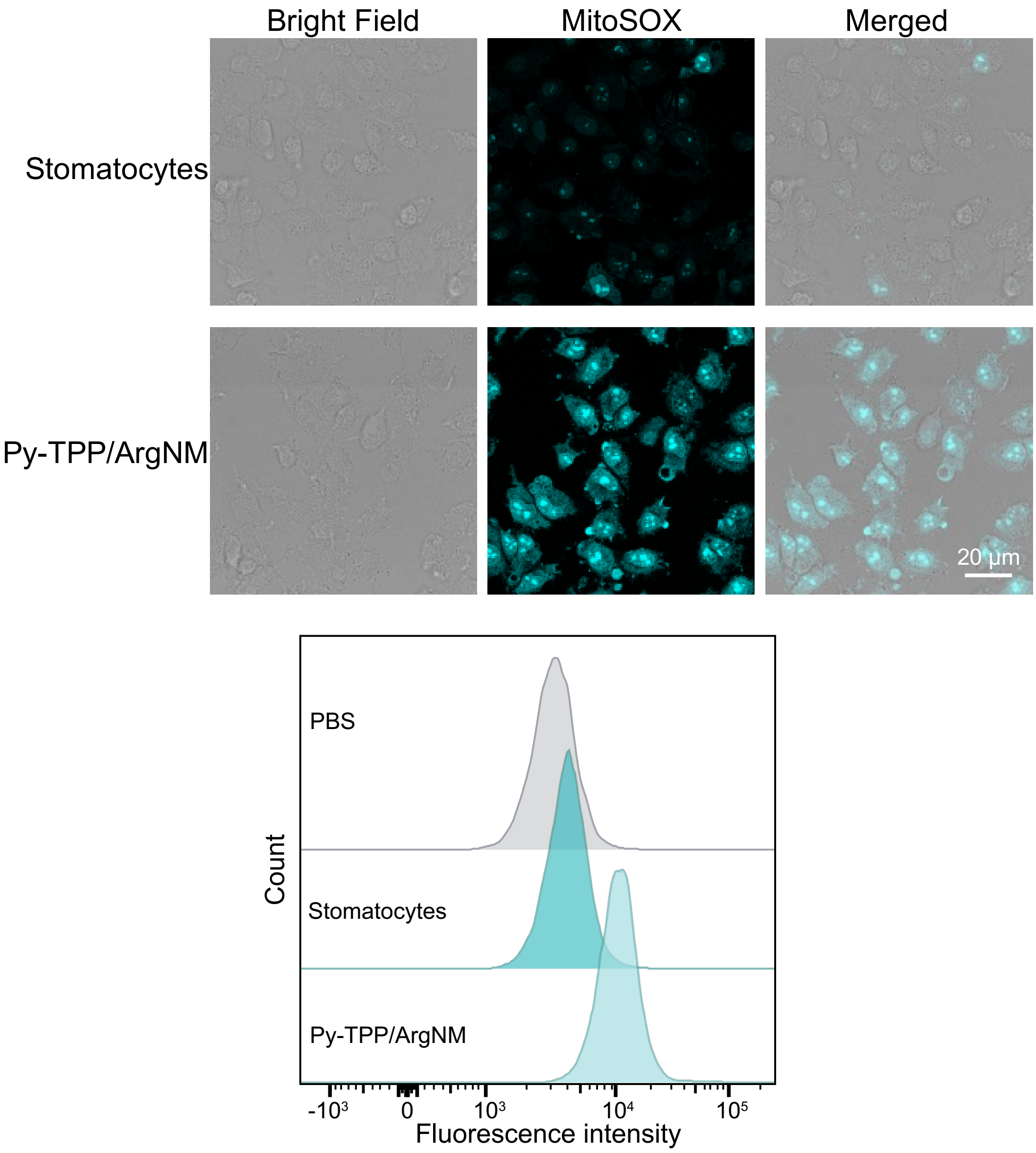

Figure S6. Fluorescence images and flow cytometry analysis of the superoxide accumulation in Hela cells.

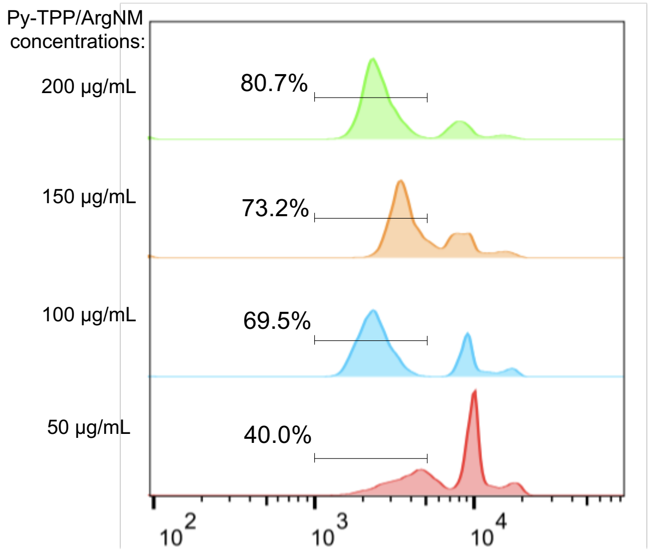

Figure S7. Cytochrome C release induced by Py-TPP/ArgNM at different concentrations.

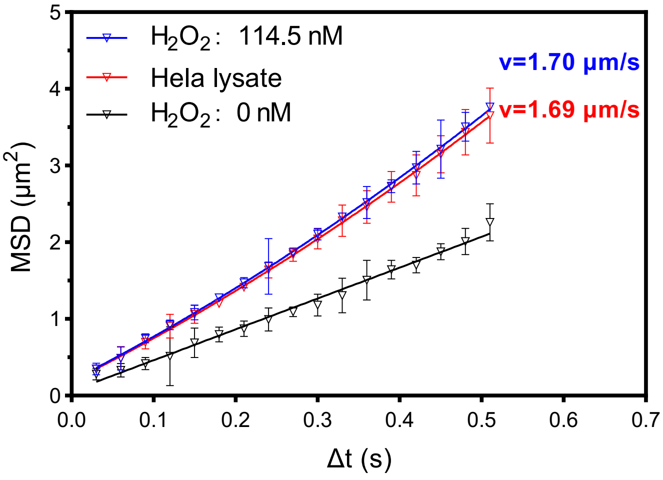

Figure S8. MSDs of Py-TPP/Arg in Hela lysate, 114.5 nM of H_2O_2 and Milli-Q water. Velocity was extracted from the fitting of the average MSD of Py-TPP/ArgNM, calculated from the tracking coordinates of, on average, 50 particles.

Table S2. Calibration curve of the oxidizing ability of H_2O_2 at different concentrations

H_2O_2 concentration (nM)	Emission at 530 nm (a.u.)
1	52
10	141
100	422
200	554
500	1028

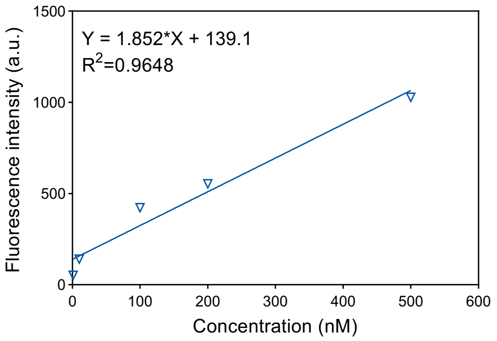

Figure S9. Calibration curve of the oxidizing ability of H_2O_2 at different concentrations

Table S3. Oxidizing ability of Hela cell lysate compared to H_2O_2

Hela cells density (cell per mL)	Emission at 530 nm (a.u.)	Equivalent to H_2O_2 (nM)
1×10^3	27	11.85
1×10^4	84	38.31
1×10^5	187	100.42
1×10^6	271	114.46
1×10^7	357	285.65

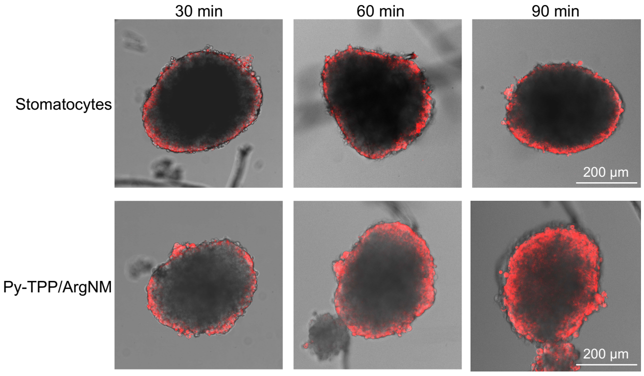

Figure S10. Fluorescence images showing the fluorescence distribution of Nile-red labeled stomatocytes or PyTPP/ArgNM.

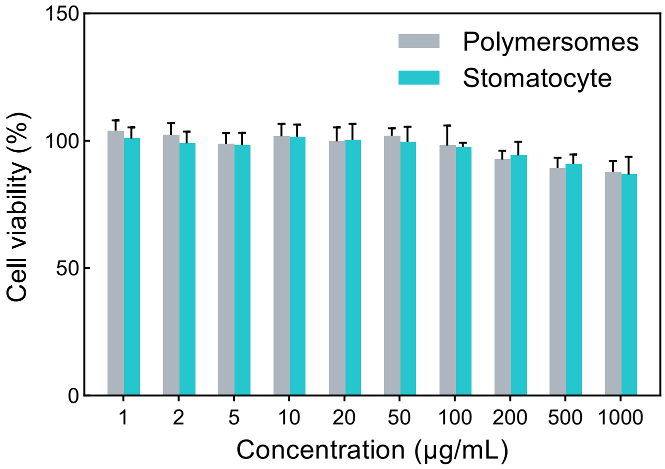

Figure S11. CCK-9 based cytotoxicity assay of the blank stomatocyte and polymersomes at varying concentrations (NIH-3T3 cells, 72 hours of incubation, 1~1000 μg/mL, calculated as the polymer materials).

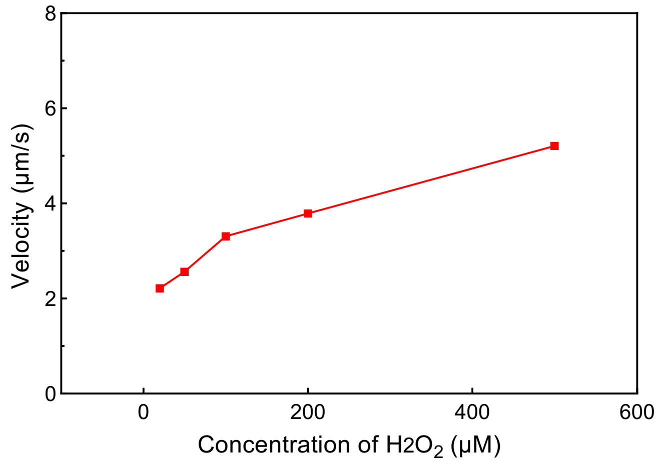

Figure S12. The velocity of Py-TPP/ArgNM in different concentrations of H_2O_2

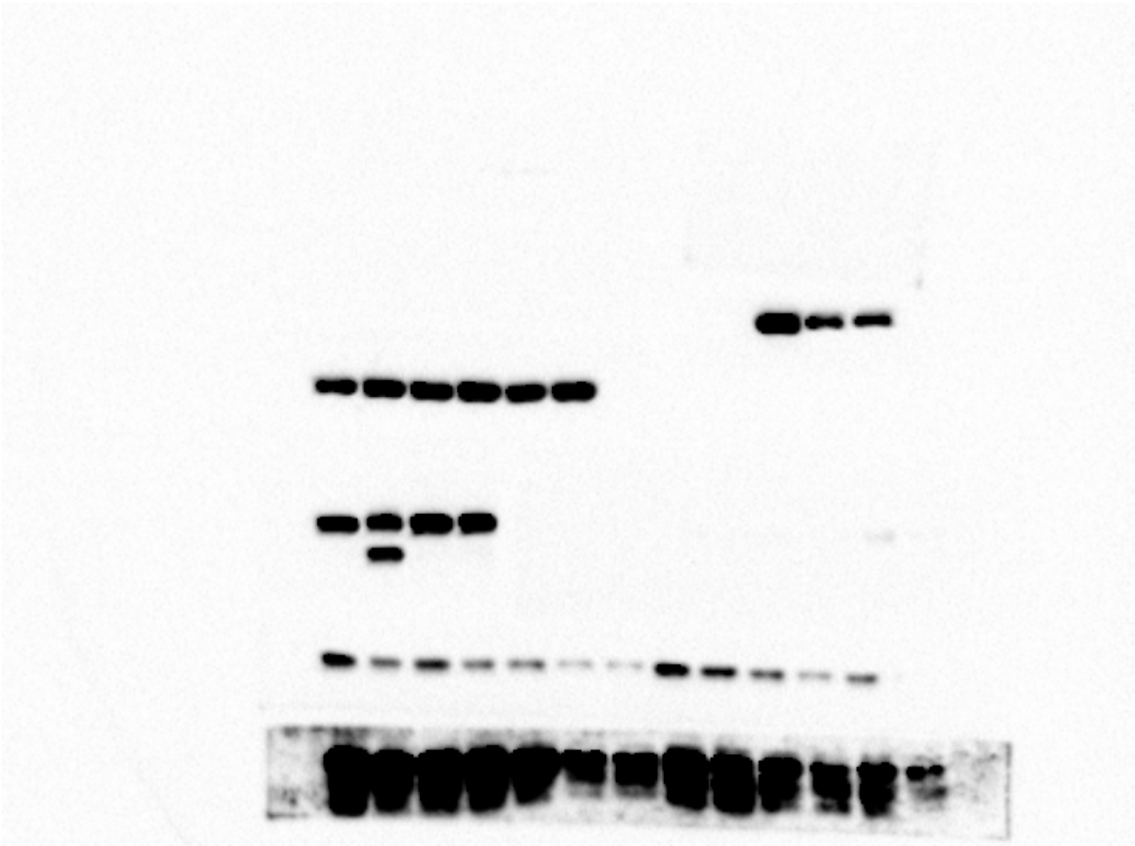

Figure S13. Uncropped/full-size gel for Figure 5c.
